# Translational medicine: An effective approach to improve healthcare by research and innovation in developing countries

**DOI:** 10.1097/MD.0000000000046355

**Published:** 2026-05-12

**Authors:** Ali Dabbagh, Firoozeh Madadi, Moein Ebrahimi

**Affiliations:** a Anesthesia Department and Anesthesiology Research Center, Shahid Beheshti University of Medical Science, Tehran, Iran; b School of Medicine, Shahid Beheshti University of Medical Sciences, Tehran, Iran.

**Keywords:** barriers, bench-to-bedside research, developing countries, translational research, translational science

## Abstract

In recent decades, medical science production has increased significantly. Cellular and molecular mechanisms of many diseases were discovered. However, the quantity of treatments and other healthcare-related technologies has not progressed significantly. As a result, translational medicine, a research approach that links bench to bedside and community, was introduced. Nevertheless, the aforementioned approach was not efficient enough to improve healthcare in a timely manner. So, translational medicine was established to discover and address prohibitory issues of translational medicine. Nevertheless, the development of translational medicine in low and middle-income countries faces numerous challenges that need to be resolved. The aim of this narrative review is to describe the recent development of translational medicine in the world and its challenges in low and middle-income countries.

## Graphical abstract

**Figure:**
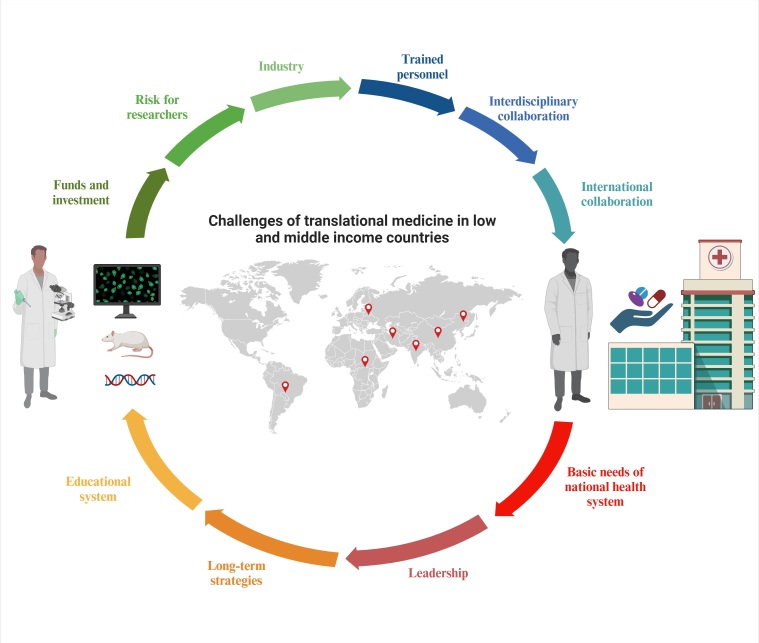

## 1. Introduction

Translational medicine is a relatively new approach to research in medicine.^[1]^ During the 1970s, translational medicine was described as based on the integration of basic sciences and clinical sciences.^[1,2]^ Translational medicine aims to study the translational process to increase its efficacy and productivity by accelerating the whole process. Translational medicine began to shape around the years 2007 to 2008, starting with the “from bench to bedside” approach. Over time, this approach transformed, leading to the fusion of basic and clinical aspects. Gradually, the triangle of “basic-clinical-community” emerged, representing the ultimate goal of medical translation.

According to the latest classification, there are 5 distinct stages in translation research, as outlined in Table 1. The stages of growth and development in medical translation were expected to become more effective and faster over the years.^[3–6]^ However, it takes approximately 15 to 20 years for a discovery to achieve community if it at all succeeds.^[1]^

**Table 1:** Stages of translational research (from bench to bedside to community).

Stage of translation	Description
T0	Basic science (discoveries in laboratories)
T1	Translation to humans (phase 1 clinical trials)
T2	Translation to patients (phase 2 and 3 clinical trials)
T3	Translation to practice (phase 4 clinical trials)
T4	Translation to the community (diffusion research)

Although translational medicine was initially introduced to improve productivity in medicine, it could not fulfill the goal, and despite increases in the quantity of research and publications, there still exists a valley of death – a valley between science and community – which deprives the community of the ability to achieve the benefits of laboratory or even clinical sciences.^[1,7]^ As a result, translational medicine, a science, was introduced: the integration of medical disciplines, reducing boundaries between medical branches, and expediting the discovery of solutions to healthcare system issues while enhancing the efficiency of medical research in addressing healthcare challenges.^[8]^ In this review, we will point out some examples of translational medicine around the world and discuss the potential benefits for our community.

## 2. Discussion

In recent years, in some countries worldwide, there has been a growing interest in medical translation to improve prevention, diagnosis, and treatment. The hope is to solve a significant portion of healthcare problems by adopting an approach that relies on the synthesis of research in various medical fields, aiming for more effective and expedited solutions to address healthcare challenges.

The National Center for Advancing Translational Sciences is a substitute of the National Institutes of Health in the United States of America. The goal of this center is to transform translational science to get more healthcare products to the community on time. National Center for Advancing Translational Sciences focuses on issues affecting the success and efficacy of translational medicine and emphasizes collecting accurate data, utilizing new technologies, and developing teamwork in favor of demonstrating and disseminating discourses that facilitate the process of translational medicine.^[9,10]^

In 2021, Christopher P. Austin published a review article about the advancements of translational medicine after a decade of taking action. They have illustrated the complexity of the whole translation process in a 4D model in order to point out the most important rate-limiting issues in translational medicine. Consequently, the scientific priorities of translational medicine in the next decade were clarified, which are summarized in Table 2. Finally, for better function, there must be a deep understanding of translation and translational medicine, as well as precise academic disciplines and academic scientific credit.^[1]^

**Table 2:** NCATS’ research agenda for the upcoming decade.

Predictive efficacy Predictive toxicology De-risking undrugged targets/currently untreatable diseases The “Chemical Space” problem New therapeutic modalities to reach currently inaccessible disease space New methodologies to increase efficiency in preclinical development Harnessing pleiotropy and promiscuity for therapeutic development Patient/community engagement Biomarkers for human clinical response Disease natural history, registries, and clinical outcome criteria Clinical trial designs Clinical trial operational efficiency Single/harmonized IRBs Clinical trial participant recruitment, retention, and diversity Clinical trial networks Data interoperability Shortening the time of intervention adoption

NCATS = National Center for Advancing Translational Sciences.

In 2010, China started developing this emerging, multidisciplinary approach to science, and more than 7 centers were established in less than a year, most of them focusing on cancer diagnosis, prognosis, treatment, acute and chronic diseases, and widespread infections. Financial support is not a major issue in China because there are many sources of investment that support potentially beneficial discoveries. However, like many other countries, the precise definition and specific goals and alignments are not well clarified.^[11]^

The European Society for Translational Medicine (EUSTM) addressed the aforementioned issue and defined translational medicine “as an interdisciplinary branch of the biomedical field supported by 3 main pillars: benchside, bedside, and community.” EUSTEM is a worldwide nonprofit organization that aims to improve worldwide health by enhancing and standardizing translational medicine. EUSTM runs many programs; the Global Translational Medicine Consortium and the Academy of Translational Medicine Professionals are among the most important. Their goal is to cross boundaries across various disciplines, resources, expertise, and technologies in the process of translational medicine in order to improve public health status. The Innovative Medicines Initiative is another TM program in Europe, aiming to enhance medicine development. EATRIS, the European Advanced Translational Research Infrastructure in Medicine, active in ten European countries, focuses on planning for translational research infrastructure in Europe and training the next generation of translational scientists.^[12,13]^

In England, biomedical research centers are working to boost translational medicine. There are multiple comprehensive and specialist BRCs, enhancing translational research in various fields of medical science. Moreover, STMTI (Wellcome Trust Scottish Translational Medicine and Therapeutics Initiative) is funded to arrange scientific teams consisting of translational medicine experts. Many translational medicine programs have already been initiated in the United Kingdom, including Master’s, PhD, and postgraduate certificates, and graduates will take part in the aforementioned TM teams.^[13,14]^

One of the most successful examples of translational medicine efficacy was seen in the COVID-19 pandemic.^[15]^ Many countries overcame the worldwide fatal event by timely preparing vaccines and treatments. Many of the boundaries were omitted, and all aspects of translational medicine, even among different countries and industries, collaborated to discover vaccines and disseminate them to the whole world with the aim of controlling the pandemic.

The COVID pandemic is over, yet this will not be the last. Moreover, there are still many unmet needs in the field of medicine. Among the approximately 800 described diseases, only a few (<600) have treatments, and many are not diagnosed properly, revealing inadequate efficiency of translational medicine.^[1,4,16,17]^ Neuroscience, cancer management, regenerative medicine, wide-spectrum infectious diseases, chronic disabling diseases, and artificial medicine are among the relatively hot topics that may have an important role in the quality of healthcare in the next few decades.

In our country, in recent decades, there has been a significant focus on quantitative research and technology development, resulting in valuable outcomes. However, there seems to be a huge gap between bench, bedside, and global village, and many experts believe that now is an ideal time for expanding qualitative dimensions in the era of research and technology. To achieve this goal, a national approach is necessary, and in this regard, having a cohesive plan is essential. By adopting such an approach, it is possible to not only continue quantitative development but also make more effective advancements in qualitative research in the field of healthcare.

One effective approach in this context is medical translation, which can have a significant and valuable impact on accelerating research in the healthcare system with a focus on the basic, clinical, and community aspects. Such an approach will ultimately expedite the progress of practical science in the healthcare system.

At the national level, a multifaceted, cohesive, and monitorable approach that encompasses all stakeholders and possesses inherent characteristics of innovation and integration can be pursued to achieve this goal.

The initial step after forming a national center would be addressing scientific research infrastructure and identifying the rate-limiting steps of translational medicine. We do have many valuable scientists in both basic and clinical science who already have worthy publications and discoveries; however, the bench can rarely achieve bedside, and if it does, reaching investigators and the community is almost impossible. So, we need to reengineer our biomedical research. To achieve such a goal, there must be effective teamwork between scientists of various fields, epidemiologists, experts in biomedical research, funders, and, most importantly, policymakers. There will be lots of workforces and barriers in translation; hence, we should also have some catalysts to enhance the process and translational scientists.

One of the well-known barriers in translational medicine is a lack of financial support. Translational science aims to improve healthcare by providing effective preventive, diagnostic, and treatment measures to the global community. It can be concluded that these 2 are directly related, so when translational science teams operate efficiently, investments in discoveries won’t go to waste, and both investors and the general population will benefit from the outcomes of collaborations. If expert translational medicine teams with knowledge of all aspects of translational medicine, including commercial policies, exist, stakeholders and industries may trust their decision-making and overcome the financial barrier between research and investment.

To prepare TM scientific teams, experts in this field must be engaged. Gilliland et al presented 7 fundamental characteristics of a successful translational scientist, namely: boundary-crosser; team player; process innovator; rigorous researcher; skilled communicator; system thinker; and domain thinker.^[18]^ Some scientists must be educated to work in the era of translational science, and some job opportunities must be defined to officially accelerate the efficiency of translational medicine. This field is relatively new in our country, and the first generation of translational scientists is being trained in postgraduate programs at some universities; however, their future job positions are still vague. In the meantime, academic anesthesiologists already have expertise in many of the mentioned domains and can be trained more quickly, so they seem to be appropriate candidates for the initiation of translational science policies in Iran.^[19]^

Anesthesiologists are naturally suited to be translational scientists due to their skills as systems thinkers and process innovators. Historically, anesthesiologists have successfully integrated basic research findings into clinical practice, leading to significant improvements in patient care, such as advancements in anesthesia safety and efficacy. This is partly because of their need for integration of physiology and pharmacology into clinical practice and decision-making. For instance, during the COVID-19 pandemic, anesthesiologists played a critical role in the rapid development and implementation of new treatment strategies, demonstrating their ability to translate scientific discoveries into practical applications.^[19,20]^ These potentials make anesthesiologists particularly valuable in translational medicine, especially in resource-limited settings like Iran, where the first generation of translational scientists has not been trained.

### 2.1. Challenges of translational medicine in low and middle-income countries (LMICs)

Although there is great potential in the field of translational medicine, low and middle-income countries (LMICs) still have a lot of progress to make in this area. In the following section of the paper, we will examine the challenges addressed in the field of translational medicine in LMICs.

#### 2.1.1. Leadership

##### 2.1.1.1. Fund distribution

Studies have revealed that fund distribution in developing countries can be influenced by personal interests and biases. In the area of translational medicine, it is crucial to have strong leadership that can make accurate choices about where to invest limited funds. It is crucial for translational medicine to thoroughly study the population without any bias and identify vulnerable subgroups in order to develop an appropriate strategy.^[21]^ In the context of LMICs, where resources are limited, there may be a strategic advantage in prioritizing clinical and community-based research to address the immediate health needs of vulnerable populations. Basic science is crucial for foundational discoveries, but its impact is limited without translation into clinical trials and community implementation. For example, funding clinical trials that target prevalent diseases in LMICs or community interventions that improve access to healthcare can yield direct benefits. Progress from basic science to human studies is costly, and insufficient funding can lead to suboptimal outcomes. Thus, a balanced approach with a slight preference for clinical and community stages could maximize impact in resource-constrained settings, ensuring that research addresses local health priorities and benefits vulnerable groups.^[22,23]^

##### 2.1.1.2. Long-term strategy

Advancing in the field of translational medicine requires significant financial investment and a long-term commitment. Therefore, it is crucial to consider the likelihood of failure when evaluating ideas in this field. This will help prevent wasted time, funds, and disappointment among researchers in this field. Effective leadership may promote a productive collaboration among government, academia, and commercial sectors. Without proper strategies and clear priorities in the field of translational medicine, it becomes challenging to develop practical ideas that can benefit society.^[21,24]^

#### 2.1.2. Government and funds

##### 2.1.2.1. Providing funds

Two crucial factors in advancing translational medicine are the support from funders and government policies. These 2 crucial elements greatly influence the trajectory of research in LMICs. Insufficient funding, lack of government organization, and limited focus on translational medicine represent significant challenges in this field.^[21]^ Research in the field of translational medicine can be quite costly. More expenses will arise the more we advance the field from basic science to human studies.^[24]^ Insufficient funding and inadequate focus in this area can have detrimental effects on research outcomes. As a result, the findings of basic science researchers may not align with the ultimate objective of conducting experiments on human subjects.^[24]^ These suboptimal outcomes fail to bring in the necessary funding, both from local government and international organizations. As a result, the issue of funding and the lack of a comprehensive government plan will persist in the future.

LMICs face significant challenges in attracting investments for translational medicine due to a combination of systemic and structural barriers. Limited government budgets often prioritize immediate short-term healthcare needs over research, leaving little funding for advancing scientific discoveries into clinical applications.^[25,26]^ Reliance on international funding exacerbates the issue, as these resources frequently align with global priorities rather than addressing local health challenges, reducing their relevance and appeal to domestic stakeholders. The high costs of translational research, including the transition from basic science to clinical trials, require substantial financial and technical resources that LMICs typically lack, deterring potential investors. Inadequate infrastructure, such as outdated facilities and a shortage of modern equipment, further undermines research quality and investor confidence. Brain drain compounds these problems, as talented researchers often leave for better opportunities abroad, depleting the local expertise needed to drive projects. Policy and governance issues, including inconsistent national strategies and biased fund allocation, create an unstable environment for investment. Additionally, cultural and economic barriers such as limited societal emphasis on research amid pressing survival needs, along with complex regulatory and ethical frameworks, increase the risks for funders. Finally, the lack of recognition and incentives for researchers diminishes motivation and fails to signal a robust research ecosystem to investors. This creates a self-reinforcing cycle where poor research outcomes discourage further investment, perpetuating the funding gap. To break this cycle, LMICs need increased domestic funding, international partnerships tailored to local needs, capacity building to strengthen infrastructure and expertise, and strategic policies to foster a supportive research environment.^[26]^

##### 2.1.2.2. Basic needs of the national health system

Tropical medicine and public health research are critical areas in which LMICs require significant support in order to prevent the demise of more individuals on the brink of mortality. It is concerning that these countries are unable to prioritize areas like translational medicine when they are struggling to meet the basic needs of their national health systems, which are crucial for preventing deaths. It is important to take into account the economic limitations associated with this issue. During occasions of limited national income, the government must establish priorities that prioritize the prevention of death over the advancement of translational medicine, which is a long-term objective.^[27]^

##### 2.1.2.3. Challenge of international funds

The majority of research funding in LMICs comes from external sources, with only minimal support from the government. Many of the funders enter with previously developed concepts that ignore the issues facing the local community. As a result, research studies that address local health issues instead of global ones tend to receive less attention from international funders.^[28]^

##### 2.1.2.4. Design of educational and employment system

Academic researchers collaborate with government officials to update legislation that facilitates academic research and clinical trials. Governments also employ numerous physicians and researchers through universities and hospitals, which has a significant impact on the availability and need for a skilled workforce. In the absence of suitable job prospects, talented individuals in LMICs may choose to immigrate to wealthier countries, leading to a loss of valuable human capital.^[28,29]^

##### 2.1.2.5. Industry system

The collaboration between academia and industry is crucial in advancing potential therapeutic interventions from basic research to clinical trials and ultimately making them accessible to the public. While universities are known for their expertise in basic research and phase 1 clinical studies, the industry has the resources required and knowledge to carry out later-phase clinical trials and effectively scale products. The employment of workers trained at universities is further supported by a strong industry presence, which in turn contributes to the growth of the economy.^[30]^

#### 2.1.3. Lack of interdisciplinary collaborations

Translational medicine remains a relatively unfamiliar concept in LMICs. In order to achieve more favorable outcomes in this area, basic science researchers must focus on creating studies that have practical potential in clinical settings, rather than solely aiming to enhance our understanding. However, clinical researchers who lack knowledge of translational medicine cannot effectively communicate the findings of basic science researchers in clinical studies. This mutual weakness presents a significant obstacle that hinders the advancement of translational medicine.^[24]^

Interdisciplinary research among experts from different fields is crucial for advancing translational medicine. By working together, these experts can generate and share new insights that benefit both individual patients and the overall health of the population. Collaborative practice involves interdisciplinary research that aims to generate new knowledge applicable to clinical practice. It is evident that the effectiveness of healthcare professionals in delivering the best possible care relies on the exchange of expertise, knowledge, and collaborative learning.^[31]^

Additionally, it has been difficult to find collaborators interested in translational medicine in LMICs, because unfamiliar fields of expertise may pose substantial barriers to the proposed research area.^[21]^

#### 2.1.4. Risk of translational medicine for researchers

##### 2.1.4.1. Current risks

There is a growing emphasis on the involvement of academic biomedical scientists in translational medicine. Nevertheless, investigators face overlooked drawbacks when their translational endeavors fail to result in favorable outcomes in clinical trials. The benefit of a novel therapy may not always be readily apparent during the initial phases of clinical development. In current times, academic researchers are frequently requested to elucidate the implications of their discoveries for human disease and to participate in experimental translational studies that illustrate the practicality of their laboratory findings for patients.^[32,33]^ Therefore, in order to encourage researchers to pursue translational medicine, it is necessary to educate them and provide an atmosphere of safety. Educational programs do not incorporate translational medicine. Such a system can not educate translational scientists.^[34]^

##### 2.1.4.2. The emergence of new metrics

Another difficulty is the use of metrics in academic institutions. Academic scientists are generally encouraged to publish their findings in prestigious academic journals. This encouragement ignores the significance of the clinical applicability of fundamental science findings. In addition, the quality of translational medicine over the long-term complicates this problem. Such a challenge necessitates a shift in strategy for developing national and academic strategies.^[24]^

#### 2.1.5. Modern high-quality equipment is not affordable in LMICs

Progress in diagnostic and treatment methods in both basic science and clinical investigation relies on cutting edge technologies. These days are truly fascinating for scientists and health professionals due to the advancements in technology. Unfortunately, these essential medical devices are often inaccessible and too expensive for many developing countries, particularly LMICs. Some countries struggle to provide the required funds for advanced pathology and laboratory equipment in clinical practice. This can be a barrier to progress in translational medicine, which is a costly field in medical research. Important advances have been made to support LMICs in addressing vaccines and immunizations, AIDS, tuberculosis, and malaria. However, it is not ideal for advancing translational medicine, which is aimed mainly at curing both communicable and non-communicable diseases. Considerable efforts have been made to help LMICs with vaccines and immunizations, AIDS, tuberculosis, and malaria. However, it is not suitable for the progression of translational medicine. It is crucial for every LMIC to include pathology and laboratory medicine as a vital part of their national plan for translational medicine. Additionally, all countries should have a well-defined national strategic laboratory plan in place.^[35]^

#### 2.1.6. Trained personnel

The lack of skilled personnel/scientists with specific expertise to undertake complex laboratory techniques in immunology, microbiology, molecular biology, and pharmacology also impedes translational medicine in LMICs. Having well-trained scientists who can conduct research and troubleshoot assays is of utmost importance, as they bring valuable expertise beyond simply operating the equipment. Put simply, it is crucial for scientists to utilize the most effective methods and state-of-the-art equipment to advance the field of translational medicine.^[28]^

#### 2.1.7. International collaboration

There is a lack of ongoing collaborations with external organizations to help facilitate the development of local translational medicine. For successful collaboration with international partners, it is crucial to have access to established laboratory spaces that have a proven history of conducting research in the relevant field.^[28]^ As previously stated, the limited availability of financial resources, the absence of long-term strategies, and the challenge of access to high-quality equipment pose significant obstacles for local scientists in LMICs. Thus, researchers from wealthier countries, who have access to the resources and infrastructure necessary for advancing translational medicine, may lack the motivation to contribute to its progress in LMICs.

### 2.2. Solutions

#### 2.2.1. Centers of excellence

A practical solution is to gather translational researchers in 1 center instead of separate researchers working in isolation. A similar idea is academic hospitals that have a lab inside or near them. Such a structure can make a close connection between basic science researchers, clinicians, and policy makers. Moreover, this strategy can maximize the output of limited resources and efforts in this field.^[36,37]^

#### 2.2.2. Training a new generation of scientists

LMICs need to invest in training a new generation of scientists to make a bridge between basic science and clinical practice. The practical examples that have happened in other countries are the MD-PhD program and the physician–scientist. Such trained scientists can fulfill the gaps between basic science and clinical practice.^[36,37]^

#### 2.2.3. Focus on local affordable needs

LMICs have achieved notable successes in translational research by focusing on practical, context-specific solutions and building local capacity. One prominent example is the development and implementation of genomics-informed interventions. In Mexico, research on asthmatic children revealed that those with a specific genotype (GSTM1 null) were more susceptible to the adverse effects of ozone on lung function. This finding was translated into a targeted intervention where antioxidant supplementation (vitamins C and E) was shown to be particularly effective for children with this high-risk genotype. Similarly, Egypt, which has the world’s highest prevalence of hepatitis C, has successfully transitioned to more effective, genomics-informed drug regimens, which have significantly improved patient outcomes. Beyond genomics, community-based approaches have also shown success.^[36]^ For instance, some LMICs, such as IRAN, have established effective newborn screening programs for congenital hypothyroidism, a condition that can be inexpensively treated with oral thyroxine.^[38,39]^ These examples demonstrate that successful translational research in resource-limited settings is often a result of focusing on local health priorities, leveraging existing resources, and developing targeted, affordable, and actionable interventions.^[36,38,39]^

## 3. Conclusion

Translational science is a nascent field in medical research, aiming to improve overall health status. This is an interdisciplinary approach across basic and clinical science, infrastructure, technologies, stakeholders, and the community, which is essential for providing new effective treatments as well as preventive and diagnostic measures to the global village in a timely manner. Our country needs to take this measure to transform the large quantity of research and publications into invaluable products.

## Author contributions

**Conceptualization**: Ali Dabbagh.

**Methodology**: Firoozeh Madadi.

**Writing – original draft**: Firoozeh Madadi.

**Writing – review & editing**: Moein Ebrahimi.
